# Pharmaceutical Pollutants: Ecotoxicological Impacts and the Use of Agro-Industrial Waste for Their Removal from Aquatic Environments

**DOI:** 10.3390/jox14040082

**Published:** 2024-10-15

**Authors:** Ana Gabriela Estrada-Almeida, María Luisa Castrejón-Godínez, Patricia Mussali-Galante, Efraín Tovar-Sánchez, Alexis Rodríguez

**Affiliations:** 1 Especialidad en Gestión Integral de Residuos, Facultad de Ciencias Biológicas, Universidad Autónoma del Estado de Morelos, Av. Universidad 1001, Col. Chamilpa, Cuernavaca C.P. 62209, Mexico; gabi_jazz95@hotmail.com; 2 Facultad de Ciencias Biológicas, Universidad Autónoma del Estado de Morelos, Av. Universidad 1001, Col. Chamilpa, Cuernavaca C.P. 62209, Mexico; 3 Centro de Investigación en Biotecnología, Universidad Autónoma del Estado de Morelos, Av. Universidad 1001, Col. Chamilpa, Cuernavaca C.P. 62209, Mexico; patricia.mussali@uaem.mx; 4 Centro de Investigación en Biodiversidad y Conservación, Universidad Autónoma del Estado de Morelos, Av. Universidad 1001, Col. Chamilpa, Cuernavaca C.P. 62209, Mexico; efrain_tovar@uaem.mx

**Keywords:** agro-industrial waste, aquatic environments, biosorption, drug pollution, ecotoxicology, pharmaceuticals

## Abstract

Medicines are pharmaceutical substances used to treat, prevent, or relieve symptoms of different diseases in animals and humans. However, their large-scale production and use worldwide cause their release to the environment. Pharmaceutical molecules are currently considered emerging pollutants that enter water bodies due to inadequate management, affecting water quality and generating adverse effects on aquatic organisms. Hence, different alternatives for pharmaceuticals removal from water have been sought; among them, the use of agro-industrial wastes has been proposed, mainly because of its high availability and low cost. This review highlights the adverse ecotoxicological effects related to the presence of different pharmaceuticals on aquatic environments and analyzes 94 investigations, from 2012 to 2024, on the removal of 17 antibiotics, highlighting sulfamethoxazole as the most reported, as well as 6 non-steroidal anti-inflammatory drugs (NSAIDs) such as diclofenac and ibuprofen, and 27 pharmaceutical drugs with different pharmacological activities. The removal of these drugs was evaluated using agro-industrial wastes such as wheat straw, mung bean husk, bagasse, bamboo, olive stones, rice straw, pinewood, rice husk, among others. On average, 60% of the agro-industrial wastes were transformed into biochar to be used as a biosorbents for pharmaceuticals removal. The diversity in experimental conditions among the removal studies makes it difficult to stablish which agro-industrial waste has the greatest removal capacity; therefore, in this review, the drug mass removal rate (DMRR) was calculated, a parameter used with comparative purposes. Almond shell-activated biochar showed the highest removal rate for antibiotics (1940 mg/g·h), while cork powder (CP) (10,420 mg/g·h) showed the highest for NSAIDs. Therefore, scientific evidence demonstrates that agro-industrial waste is a promising alternative for the removal of emerging pollutants such as pharmaceuticals substances.

## 1. Introduction

The world population recently surpassed 8000 million people, distributed mainly in Asia and Africa [1,2]; however, at a slower rate, it is projected that the global population will continue to grow in the coming years, a fact that implies important environmental, social, and economic challenges [3,4]. The accelerated increase in the world’s population generates high pressure on natural systems, given that a growing population demands more resources from the environment, including water, soil, energy, and, of course, food. As a result, various materials that serve as inputs for different industries may be overexploited [5]. Likewise, a constantly growing population demands more urban infrastructure and services and the development of efficient health systems to address the challenges derived from the growing incidence of infectious diseases, chronic degenerative diseases, and those associated with climatic situations or environmental pollution [6,7].

As a result of advances in medical knowledge about the causes and consequences of various pathologies, as well as knowledge of the therapeutic effects of substances of natural origin produced by microorganisms, fungi, plants, and even animals, different drugs have been developed for the treatment of diseases [8,9], currently, drugs are an integral part of global health systems [10]. However, a population ever-increasing in number requires the production of drugs in higher quantities to guarantee their use in health and disease treatment, generating the current growth in the pharmaceutical market [11].

However, the economic development of the pharmaceutical products industry results in a high environmental impact associated with its large-scale production and use [12,13], releasing different pharmaceutical molecules into the environment, which are currently considered emerging pollutants [14,15]; thus, their environmental presence is increasingly common around the world [16,17,18].

The presence of these molecules in the environment is recognized as a potential risk for organisms and human health, which is why they are classified as emerging pollutants due to (1) their environmental presence being increasingly evident, mainly in water bodies; (2) conventional treatments for wastewater treatment generally not effectively eliminating these contaminants; (3) their adverse effects on the environment and human health, despite some progress made, not yet being fully understood; and (4) the regulation of their environmental release and the permissible concentration limits in water bodies being in its early stages due to a lack of information on these molecules’ adverse effects [19,20,21,22].

Because of the potential risks derived from the presence of these contaminants in water bodies, different alternatives have been proposed for their elimination from aquatic ecosystems, which include physical, chemical, biological, or hybrid options [23], as well as the use of non-conventional materials for their removal, among which different lignocellulosic materials generated as waste during agroindustry production processes are pointed out. These wastes stand out because they are materials that are not used or reused largely in agricultural practices, thus promoting their high availability and low cost [24,25,26].

This review includes recent studies on the ecotoxicological effects of pharmaceutical pollutants (antibiotics, non-steroidal anti-inflammatory drugs (NSAIDs), hormones, and psychotropic pharmaceutics) on aquatic environments. These studies highlight the acute and chronic adverse effects of these pharmaceutical molecules on different organisms, including microorganisms, microarthropods, mussels, fishes, and amphibians.

This review also includes recent original studies on the use of residual lignocellulosic materials generated in the agroindustry to remove contaminants of pharmaceutical origin from water. The present review aimed to identify the most studied contaminants of pharmaceutical origin and the lignocellulosic materials most frequently proposed for removing pharmaceutical pollutants from water, their removal efficiency, and the treatments used to improve their contaminant absorption characteristics, mainly their conversion to biochar.

Due to the diversity in pharmaceutical molecules, agro-industrial wastes, and experimental conditions used in the studies, it is difficult to identify the agro-industrial materials with higher efficiency in removing pharmaceutical pollutants, an important drawback in this study field. In the present review, we propose the drug mass removal rate (DMRR) as a novel comparative parameter to identify agro-industrial wastes with a higher potential for pharmaceutical pollutant removal from water. The present review is helpful for the research and development of efficient and affordable pharmaceutical contaminant removal systems based on the use of residual lignocellulosic materials.

## 2. Methodological Approach

### 2.1. Information Search

In the present review, a search was carried out for original scientific studies on the use of agro-industrial waste to remove drugs in aqueous systems. The search of studies covered the period from 2012 to 2024 and was conducted in the databases (1) Google Scholar, (2) ScienceDirect, (3) SpringerLink, (4) PubMed, and (5) SCIELO using the keywords agro-industrial wastes, biomass, biosorption, drug, removal, pharmaceutical, and water. Search equations were constructed with combinations of the keywords mentioned above. Documents were initially filtered through the analysis of the title, abstract, and conclusions, and studies selected were those that met the following inclusion criteria: (1) studies published within the established period (2012–2024); (2) evaluated the removal of pharmaceutical molecule/s using agro-industrial wastes in aqueous system; and (3) included at least the following information: the initial concentration of the removed pharmaceutical molecule/s, the removal percentage/final concentration of the pharmaceutical molecule/s in the system, the contact time, and the mass/concentration of biosorbent material employed. All duplicated documents and those not meeting the inclusion criteria were excluded.

### 2.2. Drug Mass Removal Rate

The diversity in pollutant molecules, agro-industrial wastes, and experimental conditions used in the identified studies make it difficult to compare and identify the waste materials with higher efficiency in removing pharmaceutical pollutants. To overcome this drawback, in the present review, we propose the drug mass removal rate (DMRR) as a novel comparative parameter. The DMRR is a numeric parameter calculated using key information related to the pharmaceutical molecule removal process, such as (1) the initial concentration of the pharmaceutical molecule in the system (C_i_), (2) the final concentration of the pharmaceutical molecule in the system (C_f_), (3) the concentration of biosorbent material employed (C_B_), and (4) the removal time (R_T_). The DMRR refers to the milligrams of the pharmaceutical molecule removed by one gram of biosorbent material in one hour, considering that materials with higher DMRR values have a higher potential for pharmaceutical pollutant removal from water. In this work, the DMRR was calculated using Equation (1).

(1) $$DMRR=\frac{C_{i}-C_{f}}{C_{B}\cdot R_{T}}$$

where

DMRR = drug mass removal rate (mg/g·h);
C_i_ = initial concentration of the pharmaceutical molecule (mg/L);
C_f_ = final concentration of the pharmaceutical molecule (mg/L);
C_B_ = concentration of biosorbent material (g/L);
R_T_ = removal time (h).

## 3. Emerging Pollutants

Every year, different xenobiotic chemical substances are released into the soil, water, and air, generating the presence of complex mixtures of chemical pollutants difficult to characterize through conventional analytical methodologies. These mixtures also show higher toxicity due to additive and synergic adverse effects and represent a challenge for their elimination from the environment [27,28,29,30]. Due to its adverse effects on natural systems and human health, environmental pollution derived from the release of different xenobiotic compounds into soil, water, and air is a global concern [31]. Extractive, agricultural, and industrial activities are recognized as the main generators of environmental pollution [32,33].

Various types of contaminants may be present in the environment; however, organic molecules such as different chemicals of industrial use, pesticides, drugs, and personal care products are increasing their frequency of detection in the environment, all together constituting a group of substances currently recognized as organic contaminants of emerging concern due to their toxicity and potential adverse impacts on ecosystems and human health [34,35,36,37,38]. To date, the development of regulations, directives, or policies to reduce pollution derived from emerging pollutants and establish the maximum allowable limits for such substances is limited in many countries [39,40].

Emerging pollutants have different biological adverse effects on organisms, induce reactive oxygen species (ROS), mutagenicity, cytotoxicity, apoptosis, tissue damage, endocrine disruption, and teratogenic effects, among others [41]. On the other side, exposure to hazardous emerging pollutants could induce harmful health effects in humans, such as alterations in the immune system, damage to the nervous system, development of allergies, cancer, and reproductive disorders [42]. Emerging pollutants include, among others, substances such as agricultural pesticides, industrial and consumer product waste, illicit drugs, personal care products, and drugs for human and veterinary use [43,44].

More than a thousand substances and their degradation products are considered emerging pollutants, according to the NORMAN Network database [45]. Punctual and diffuse emission sources related to anthropogenic activities, including agriculture, industry, hospitals, and domestic, release different emerging pollutants to soil and water [46], generating severe adverse impacts on the environment, mainly in aquatic ecosystems [47]. Among emerging pollutants, pharmaceutical origin pollutants have great relevance [48].

The presence of pharmaceutical molecules in water has been related to different impacts on aquatic organisms; for example, the occurrence of different antibiotic compounds (aminoglycosides, β-lactams, fluoroquinolones, glycopeptides, macrolides, sulfonamides, tetracyclines, and trimethoprim) in aquatic environments has been related to the development of antibiotic resistance genes in microbial communities, but also toxicity on different aquatic organisms, such as cyanobacteria, algae, crustaceans, and fishes, among others [14,49]. On the other hand, NSAIDs, hormones (estrogens, progesterone, androgens, glucocorticoids, and growth hormones), and psychoactive medications, among others, are frequently identified in aquatic environments as result of industrial, hospital, domestic, or husbandry release of residual waters [50,51,52,53].

## 4. Occurrence of Pharmaceuticals as Pollutants in the Environment

### 4.1. General Information about Pharmaceutical Molecules

Pharmaceutical drugs are substances of natural or synthetic origin with biological activities capable of inducing a reaction or change in cellular or tissue functioning in organisms. These molecules treat different pathologies in animals and humans, and so are widely used in veterinary and human medicine [54,55]. Medicines are pharmaceutical products with applications in preventing, diagnosing, and treating diseases. Medications include two main components in their formulation: (1) the active or pharmacological ingredient, which refers to the molecule or molecules responsible for the pharmacological effect or biological action, and (2) the excipient, which is a substance or group of substances that facilitate the administration of the active ingredient [56,57]. Excipients include coloring molecules, emulsifiers, solvents, diluents, flavorings, preservatives, or components that regulate the release, improve the absorption, or favor the distribution of the pharmacological ingredients [58,59,60]. While pharmaceutical active principles can be classified according to their biological effects as anti-inflammatory, analgesic, antiviral, anticancer, antianxiety, antidepressant, antipsychotic, antihypertensive, antibacterial, antiarrhythmic, and diuretic, among others [61,62].

### 4.2. Pharmaceutical Molecules as Contaminants

Contaminants of pharmaceutical origin such as perfluorinated compounds, hormones, illicit drugs, personal care and hygiene products, as well as antibiotics, disinfectants, antivirals, anticancer and non-steroidal anti-inflammatory drugs, and analgesics, among others used for the treatment of diseases in humans and animals, are detected with increasing frequency in surface and underground water bodies in different countries [48,63,64,65]. These molecules have only recently been considered as environmental contaminants, and the magnitude of their adverse effects on the environment and human health have not been fully elucidated; thus, in many countries, regulations to limit their presence in the environment are still developing [66,67].

Further advances in the establishment of a regulatory framework to reduce the presence of emerging pollutants in the environment and human exposure have been made in countries such as the United States (US-EPA, Cleaner Water Act, and Safe Drinking Water Act), European Union (Water Framework Directive), Japan (Drinking Water Quality Standards), Australia (The National Water Quality Management Strategy), and Canada (Environment and Climate Change Canada). Establishing stricter regulations on emerging pollutants, including drugs and personal care products, has reduced the number of unregulated polluting molecules. In a more global attempt to regulate the release of several pollutants to the environment, including pharmaceutical pollutants, due to irrigation with wastewater in agricultural practices, the World Health Organization established the Guidelines for the Safe Use of Wastewater. However, regulatory advances in other countries have been limited [22,68,69,70].

To the extent that pharmaceuticals are used in greater frequency and quantities by the population, the environmental presence of drugs, their residues, and metabolites becomes evident [71]. Due to their persistence, these compounds can remain in the soil and surface waters for extended periods, generating environmental impacts in various ecosystems. To reduce the ecological presence of contaminants of pharmaceutical origin, different wastewater treatment strategies and the remediation of these sites have been proposed, allowing for the quality of water bodies to be improved and limiting their impact on human health.

**Figure 1:**
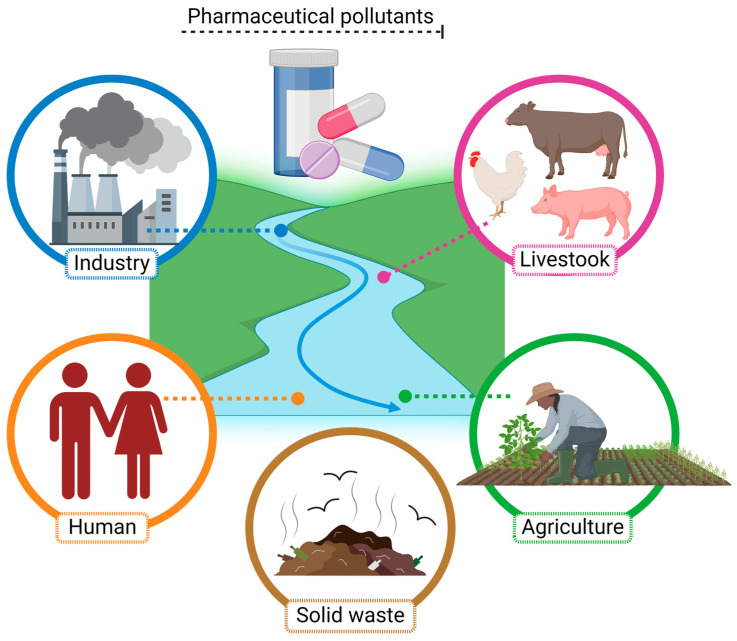
Sources of pharmaceutical environmental pollution.

Pharmaceutical molecules can reach the environment through different pathways (Figure 1). The most representative include (1) environmental release after use in human and veterinary medicine: pharmaceutical molecules used as therapeutics are excreted as the original molecules or derived metabolites reaching wastewater; (2) inadequate management of expired drugs in homes driving inadequate disposal into domestic solid wastes and directly to the drainage system, also reaching soil and water; (3) wastewater treatment systems being unable remove all pharmaceutical molecules present in wastewater, enabling pharmaceutical molecules to reach natural water bodies or be concentrated in sewage; and (4) pharmaceutical molecules, such as antibiotics and hormones, often being used in agriculture and livestock: after their use, these molecules reach soil and water [72,73].

The environmental presence of pharmaceutical molecules induces adverse effects on the environment, both in soil and water. In aquatic systems, such as rivers and oceans, pharmaceuticals can affect the reproduction rates and development of different aquatic species (algae, mussels, fish, and mammals) due to their toxicity and endocrine disruption characteristics. Moreover, pharmaceutical molecules can be bioaccumulated in the organism’s tissues and biomagnified through the food chain, affecting population dynamics and threatening biodiversity [74,75,76]. Exposure to pharmaceutical molecules in drinking water can also affect human health, even at low doses, causing several acute and chronic effects due to sustained exposures [77,78].

Due to the adverse environmental and human health effects of pharmaceutical pollutants, it is necessary to establish monitoring systems to detect their presence in the environment and regulatory issues to reduce their release and environmental concentration levels, a complex task due to the vast diversity in these molecules, their sources, adverse effects, and the pathways through which they can reach the environment. Hence, the development of effective and low-cost technologies to remove these pollutants from water can help to reduce their environmental presence and mitigate human health risks [24,79,80].

## 5. Ecotoxicological Effects of Pharmaceutical Pollutants in Aquatic Environments

**Figure 2:**
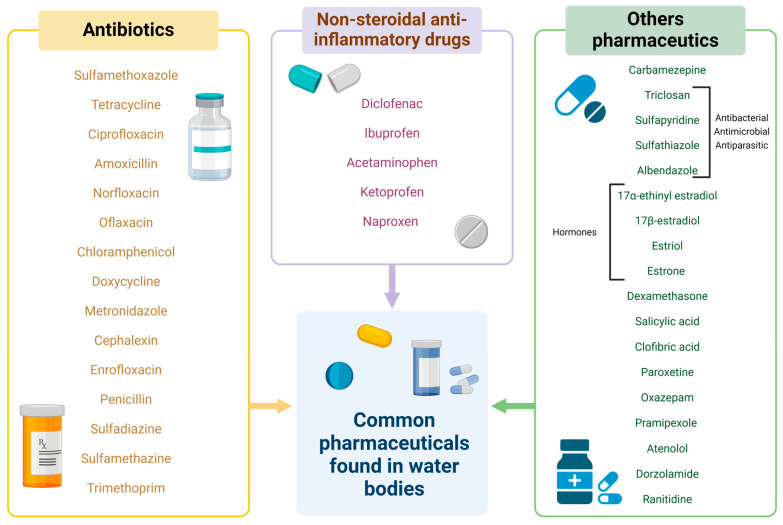
Common pharmaceutical pollutants found in water bodies.

Pharmaceuticals can generate various negative impacts on ecosystems, so it is important to monitor their presence and ecological effects in different environmental matrices, mainly soil and water [81]. Through pharmEcovigilance, a term proposed by Daughton and Ruhoy in 2008 [82], we can establish strategies to identify the presence of pharmaceutical pollutants in the environment and their impacts. The pharmEcovigilance approach aims to unify environment healthcare, protection, and remediation through monitoring the environmental presence of pharmaceutical pollutants, identifying the main routes of exposure, characterizing the short- and long-term ecological impacts derived from their environmental presence, and establishing measures to reduce their accumulation in the environment [82]. Different pharmaceutical molecules, including various classes of antibiotics, NSAIDs, hormones, and many others, can pollute water bodies (Figure 2), causing a plethora of adverse effects on organisms. Below are some studies that exemplify the individual and ecological impacts of pharmaceutical pollutants.

### 5.1. Antibiotics

Antibiotics are among the most used drugs worldwide due to their great importance for the prevention, control, and treatment of infectious diseases in animals and humans. As a consequence, their presence is frequently identified in terrestrial and marine environments because of the release of wastewater originating in urban areas, hospitals, and pharmaceutical industry, as well as the use of antibiotics in animal husbandry and aquaculture [83,84]. Antibiotics from different chemical families, such as beta-lactams, diaminopyridines, fluoroquinolones, macrolides, sulfonamides, and tetracyclines, as well as their degradation metabolites, occur in groundwater, rivers, and oceans, in concentrations ranging from ng/L to even mg/L. The environmental presence of antibiotics has been related to toxic effects on aquatic organisms, including various species of marine bacteria, cyanobacteria, algae, crustaceans, and fishes [85,86].

Derived from their chemical diversity and mechanisms of action, toxic effects of exposure to antibiotics are diverse; for example, exposure to antibiotics reduces the growth of algal species [87] and interferes with chlorophyll biosynthesis [88]. In a study with an ecotoxicological approach, Isidori et al. (2005) [89] evaluated the acute and chronic effects of six antibiotics (clarithromycin, erythromycin, lincomycin, ofloxacin, oxytetracyclin, and sulfamethoxazole) on different aquatic organisms such as bacteria, algae, rotifers, microcrustaceans, and fish. All antibiotics evaluated presented acute effects at concentrations in the mg/L range; however, while chronic effects occurred at lower concentrations (µg/L), the antibiotic ofloxacin presented genotoxic effects and the antibiotics sulfamethoxazole, ofloxacin, and lincomycin presented mutagenic effects in bacteria [89].

In fish, exposure to antibiotics has been related to genetic, biochemical, and morphological effects; the main biomarkers of antibiotic damage in fish include (1) genotoxicity, evaluated mainly through the comet assay and the presence of micronuclei in erythrocytes; (2) oxidative stress, through the evaluation of the expression levels in genes related to the production of antioxidant enzymes, as well as the evaluation of their enzymes; (3) neurotoxicity, through the evaluation of acetylcholinesterase (AChE) activity; (4) heart and liver damage; and (5) effects related to egg hatching levels, development, and growth, or even (6) mortality [90,91]. However, among the most documented and worrying effects of antibiotic contamination, the development of antibiotic resistance genes and mechanisms in bacteria present in the environment stands out [92,93,94].

### 5.2. Non-Steroidal Anti-Inflammatory Drugs (NSAIDs)

Non-steroidal anti-inflammatory drugs (NSAIDs) comprise a group of medications widely used in medicine during the treatment of multiple diseases because they present analgesic, anti-inflammatory, and antipyretic properties through the inhibition of the cyclooxygenase COX pathway, resulting in blocking of prostaglandin and thromboxane synthesis [95,96]. The first NSAID marketed was aspirin (acetylsalicylate); however, currently, NSAIDs such as ibuprofen, diclofenac, fenoprofen, naproxen, and oxaprozin (aryl or heteroaryl acetic acid molecules) are among the most popular worldwide [97]. In addition to their high prescription rate [98], many NSAIDs are available over the counter, so their rate of consumption and presence in the domestic environment is very high [99], resulting in high levels of these compounds and their degradation metabolites in wastewater and water bodies around the world, with concentrations reaching the order of mg/L [100,101,102,103,104]. NSAIDs impact biodiversity in wetlands, surface water bodies, and marine environments [105,106,107].

Batucan et al. (2022) [108] conducted a review of studies related to the evaluation of the toxicity and adverse effects of NSAIDs such as ibuprofen and diclofenac in aquatic organisms in the short and long term. In general, exposure to these contaminants generates adverse effects in organisms such as algae, crustaceans, fish, and amphibians, with zebrafish (*Danio rerio*) and *Daphnia magna* being the most used organisms in ecotoxicological studies with diclofenac and ibuprofen. The studies carried out evaluate the toxicity and adverse effects using various biomarkers (neurotoxicity and defense against oxidative stress, among others), physiological indicators, mortality increase, growth inhibition, morphological changes, alterations in behavior, and modifications in the community structure of aquatic environments [108].

Due to its lipophilic characteristics and low biodegradability, ibuprofen can bioaccumulate in different organisms in aquatic environments, such as mollusks, crustaceans, and fish, identifying genetic, cellular, and tissue damage; oxidative stress; effects such as lipid peroxidation; disruption of biological activities and important enzymatic activities; neurotoxicity; dysbiosis; malformations; and reductions in egg hatching rates, among many others [109]. The median lethal concentrations (LC_50_) of ibuprofen in acute exposures (15 min–96 h) for aquatic organisms were 0.4–175.6 mg/L, while chronic effects (1 week to 4 months) occurred at concentrations of 0.0003–33 mg/L depending on the organism evaluated [110].

Diclofenac and acetaminophen are two NSAIDs of great relevance, widely used to control pain and in the treatment of inflammatory disorders. Both molecules can generate adverse effects in different aquatic organisms. In the case of diclofenac and its degradation metabolites, the induction of oxidative stress and genetic damage in organisms such as bacteria, algae, mollusks, crustaceans, fish, and amphibians, among others, has been reported at concentrations in ranges from 0.4 µg/L to 4 mg/L [111,112,113,114]. The adverse effects of acetaminophen (paracetamol) on aquatic organisms have been evaluated in mollusks, polychaetes, crustaceans, and fish. Its adverse effects include alterations in biochemical, metabolic, and cellular processes; these effects occur in concentration ranges from 0.1 mg/L to 5 g/L [115,116,117].

Naproxen is one of the most used NSAIDs worldwide; it is commonly found in water bodies, including drinking water, in ranges from ng/L to µ/L. The adverse effects of naproxen and its degradation metabolites in non-target organisms include toxicity in aquatic organisms like bacteria, algae, rotifers, crustaceans, and fish. In green algal species, reduced growth, low chlorophyll and carotenoid production, presence of ROS, and a decrease in antioxidant enzymes such as superoxide dismutase (SOD) have been observed, as well as genotoxicity. In fish, naproxen bioaccumulation and liver and heart damage have been detected in fish such as zebrafish (*D. rerio*), as well as reductions in the reproductive rates of freshwater fish such as *Jordanella floridae* [118].

In a study using the Mediterranean mussel (*Mytilus galloprovincialis*) as an indicator organism, exposure to five NSAIDs—acetaminophen, diclofenac, ibuprofen, ketoprofen, and nimesulide (25 µg/L)—under controlled laboratory conditions resulted in the bioaccumulation of significant diclofenac (14.9 ± 7.89 ng/g dw), ibuprofen (1.63 ± 1 ng/g dw), and nimesulide (30.22 ± 13.5 ng/g dw) concentrations. At the same time, wild organisms collected in the field (Portonovo Bay, Italy) presented higher bioaccumulation levels of diclofenac (16.11 ± 14.72 ng/g dw) and ibuprofen (9.39 ± 0.59 ng/g dw) in comparison to those exposed to laboratory conditions. As a result of the exposure and bioaccumulation of these contaminants, different adverse effects were presented: (1) at the immunological level, a reduction in the stability of the lysosome membrane; (2) at the metabolic level, reductions in the activity of the acyl-CoA oxidase enzymes; (3) in defense against oxidative stress, ketoprofen and nimesulide reduced the activity of catalase, and ibuprofen reduced the activity of glutathione S-transferases; (4) concerning neurotoxicity, acetaminophen reduced the activity of the enzyme acetylcholinesterase (AChE); and (5) regarding to genotoxicity, all NSAIDs induced higher micronuclei frequencies while diclofenac and ibuprofen increased DNA strand breaks. In general, exposure to NSAIDs generated important adverse effects in M. galloprovincialis, so this organism was proposed for the biomonitoring of contamination by NSAIDs in marine environments [119].

The ecotoxicological effects of the environmental presence of NSAIDs are evident; acute and chronic effects of exposure to these types of drugs have been reported on different organisms that inhabit aquatic environments. In animals such as mollusks, echinoderms, crustaceans, and fishes, the adverse effects derived from exposure to NSAIDs include genetic damage, oxidative stress, metabolic disorders, endocrine disruption, teratogenesis, reductions in reproductive rates, low weight, and body deformations [103,120].

### 5.3. Hormones

Hormones are used as pharmaceuticals for treating pathologies and for birth control. Some examples are the thyroid hormones thyroxine (T4), thiioiodothryronine (T3), and their analogues, used in treatment of hypothyroidism and other thyroid function disorders [121,122]; the insulin protein hormone, in the treatment of diabetes type 1 and 2 [123,124,125]; corticosteroids such as cortisone, betamethasone, dexamethasone, prednisolone, and prednisone, among others, for the treatment of inflammation, asthma, arthritis, and autoimmune diseases [126,127]; and more commonly sex hormones such as estrogen, progesterone, and testosterone, which are used for the treatment of different medical conditions including menstrual disorders [128,129], menopause [130,131], hypogonadism [132,133,134], cryptorchidism [135,136], hormone-dependent cancers such as breast, ovary, uterus, and prostate [137,138,139], and mainly the use of estrogens and progestin as a birth control method [140,141]. As a result of their large-scale use, hormones are released into the environment, reaching water bodies. High quantities of natural (estrone, 17β-estradiol, 17α-estradiol, estriol) and synthetic (17α-ethinyl estradiol) steroidal estrogen molecules are released to the environment every year due to chemical anticonception practices, generating pollution in water bodies; several steroidal estrogens have been identified in rivers in many countries worldwide [142,143].

On the other side, during livestock production, different hormones are employed as growth promoters; these molecules can be used as additives in feed or used as veterinary drugs for improving nutrient utilization efficiency, accelerating animal growth, fattening, increasing meat and milk production, reproductive purposes, or treating inflammatory diseases [144,145]. Due to the increasing demand for animal-origin food and products by the constantly growing human population, the use of hormones in farm animal breeding is significantly rising. The most employed hormones in livestock include somatotropins (growth hormones), thyroxines (metabolic regulators), glucocorticoids, gestagens (pro-gestational effects), androgens (anabolic steroids) [146], and estrogens [146,147,148]. The use of hormones in large quantities during animal breeding generates environmental pollution. Wastewater generation in farms, which carry on urine and manure, can contain hormones that may enter water bodies through runoff or leaching into groundwater [149].

Hormones can arrive in ecosystems, cause water quality reduction, and affect aquatic life [150,151]. The presence of hormones induces important adverse effects on organisms living in these environments. Many hormones can be bioaccumulated in organisms and biomagnify through trophic levels; bioaccumulated hormones can act as endocrine disruptors in animals and humans, resulting in severe alterations in reproductive processes or in animal behavior. Hormones such as estrogens can alter the reproductive cycles in fishes, affecting fertility or potentially causing feminization in male fishes [152,153].

In aquatic crustaceans (*D. magna*), exposure to hormones (estrogens and 17ß-oestradiol) caused immobilization and changes in the molting frequency [81]. Fish species are sensitive to the presence of hormones in the environment; exposure to steroid hormones in zebrafish generates endocrine disruption, gonad damage, and sex ratio alterations [154]. The presence of steroid hormones (androgens and estrogens) in aquatic environments affects the development and generates changes in sexual characteristics of fishes. Estrogens such as estrone, 17β-estradiol, and 17α-ethinylestradiol induce feminized properties in exposed male fish [155]. Moreover, in aquatic environments, the presence of gestagens (progesterone and progestins) generates adverse effects in fish—mainly reproductive, developmental, and behavioral—because they can interfere with steroid hormone receptors [156].

It is necessary to understand the long-term impact of hormone exposure in wild animals, as well as in humans. This understanding is vital for establishing regulations to reduce the release of hormones derived from their use in human medicine, birth control, agricultural, and animal breeding practices. Equally important is the urgent need to improve the wastewater treatment systems to ensure better removal of these potentially harmful molecules before they are reintroduced into natural water bodies.

### 5.4. Psychotropic Drugs

Other pharmaceutical molecules, such as psychotropic drugs, have also been detected in water bodies; these molecules are commonly used in the treatment of stress, anxiety, depression, and epilepsy, among other mental health conditions. Drugs such as carbamazepine and diazepam, fast-action tranquilizers of the benzodiazepine family, have been detected in wastewater, treated waters, and superficial waters, in concentrations of the ng/L order. As a result of their effect on the central nervous system, exposure to carbamazepine and diazepam generates adverse effects in aquatic organisms, including direct toxicity, oxidative stress, endocrine disruption, and reproductive and behavior modifications [157].

Nogueira and Nunes (2022) [158] evaluated the effect of acute (96 h) and chronic (28 days) diazepam exposure on the polychaete *Hediste diversicolor*. Acute exposure induced alterations in behavior and hyperactivity, while hypoactivity was observed during chronic exposure. Moreover, the determination of biochemical biomarkers showed an alteration in the production of antioxidant enzymes (increase in catalase (CAT) and glutathione-S-transferase (GST) activity) and neurotoxicity (reduction in AChE activity).

Diazepam exposure causes physiological adverse effects and alterations in the swimming behavior of zebrafish embryos and male adults [159]. More recently, behavior modifications as a consequence of diazepam exposure were reported in both female and male zebrafish. These effects included a decrease in swimming velocity and locomotor activity, sedative effects, and alterations in social interaction and courtship behaviors; the adverse effects of diazepam were more significant in females [160].

In other fish species, the adverse effects of diazepam exposure have also been documented. In *Oryzias latipes* (Japanese medaka), its bioaccumulation in brain and gonad tissues was observed; diazepam exposure reduced swimming velocity, active motility, and exploration behaviors, while inducing changes in social interactions and courtship behaviors associated with differentiated changes in neurotransmitter levels between female and males. In females, dopamine (DA) and serotonine (5-HT) neurotransmitters increased their levels, while in males, the levels of γ-aminobutyric (GABA) acid were reduced [161]. In *Ictalurus punctatus* (channel catfish) exposed to diazepam (1 mg/L, 7 days), exposed fish bioaccumulated the drug in the liver, brain, muscle, gonads, and plasma; the levels of steroid hormones did not show changes in tissues, but important genes implicated in the production of different steroid hormones were downregulated [162].

Baali and Cosio (2022) [163] reviewed the adverse effects of carbamazepine on aquatic organisms (anemones, mussels, crustaceans, and fishes, among others). Overall, exposure to this drug induces oxidative stress, adverse effects on ROS homeostasis, and reductions in cell viability; alters the function of the endocrine, neurologic, and immunologic systems; and causes changes in feed behavior and reductions in growth rates [160]. Desbiolles et al. (2020) [164] evaluated chronic carbamazepine exposure (100 mg/L, 14 days) in two model organisms, *Lemna minor* L. (duckweed) and *Hydra circumcincta* (hydrozoa, cnidarian). Chronic carbamazepine exposure caused changes in nitrogen balance and the chlorophyll indices in duckweed, while lipid peroxidation and effects on morphology and reproduction were observed in H. circumcincta [164].

Bivalve mussels have been used as bioindicators of the presence of carbamazepine and for the determination of its toxic effects on marine environments; carbamazepine induces oxidative stress, genotoxicity, cytotoxicity, and genotoxicity in these organisms [165]. Carbamazepine has been broadly evaluated using different fish species as ecotoxicity models in aquatic environments. In *Carassius carassius*, carbamazepine exposure (10 µg/L, 7 days) induces neurotoxicity, reduction in acetylcholinesterase activity in the brain, and activation of antioxidant defense mechanisms [166].

Chronic carbamazepine exposure (1.25–5 mg/L, 7–21 days) causes hematological effects, such as reductions in red and white blood cells and lymphocyte counts in *Cyprinus carpio* [167]. In *Gambusia affinis* (mosquitofish), the carbamazepine median lethal concentration was 24 mg/L, while exposure at sublethal doses reduced growth and caused changes in behavior and neurotoxicity [168].

**Figure 3:**
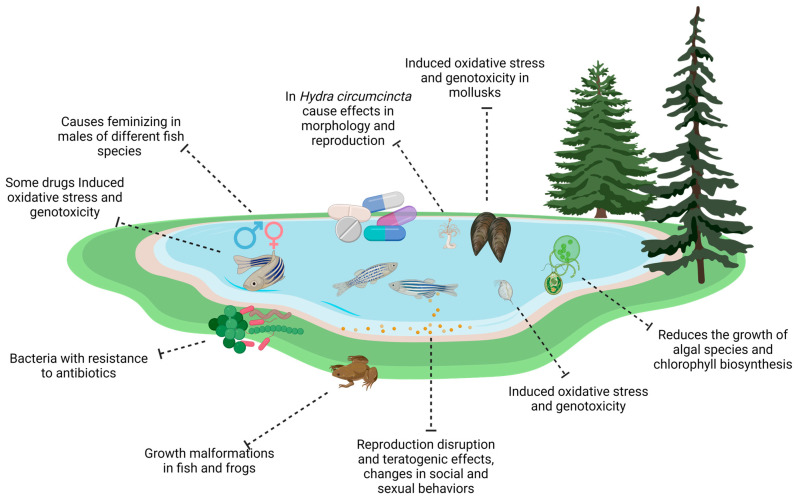
Ecotoxicological impacts of pharmaceutical pollution in aquatic environments.

The presence of pharmaceutical pollutants in aquatic environments pose a high risk for organisms living in these ecosystems, not only for their direct toxicity, but also relating to their side-effects and the adverse consequences, such as developmental and reproductive, derived from chronic exposure. Adverse effects of pharmaceutically originated pollution can be observed at different trophic levels, e.g., microorganisms, small crustaceans, mussels, different fish species, and amphibians (Figure 3). However, due to the diversity of the chemical structures and the pharmacological and biological activities of all pharmaceutical pollutants included in the present review, it is difficult to compare them in terms of toxicity or ecological impact.

The toxic effects of exposure could differ based on the chemical characteristics of each pharmaceutical pollutant, its environmental concentration, the toxic dose, the time of exposure (acute or chronic exposure), the exposure pathway, and the specific organisms under study. On the other hand, the extent of the environmental impact could also be different for each pharmaceutical pollutant due to facts such as the frequency of pollution events, its environmental concentration, its bioavailability in aquatic environments, its environmental half-life, as well as parameters such as the bioaccumulation and biomagnification potential of each pharmaceutical pollutant. Due to this, each pharmaceutical pollutant needs adequate characterization of its toxicity and ecological risk profile, highlighting the need for conduct more ecotoxicological studies employing multiple biomarkers in representative organisms of the different trophic levels. Due to their significant ecotoxicological impacts, feasible alternatives must be developed that allow for the elimination of contaminating molecules of pharmaceutical origin from aquatic environments.

## 6. Pharmaceutical Pollutant Degradation and Removal Strategies

Given the adverse ecotoxicological effects on the environment and the threats to human health derived from environmental pollution by pharmaceutical molecules, there is necessity for the development and implementation of strategies to remediate impacted sites through degradation or removal of these pollutants, with the aim of mitigating their impacts. Several strategies have been proposed to eliminate pharmaceutical pollutants, and among the most studied strategies to remove them from water are advanced oxidation processes, ionic exchange resins, chemical precipitation, electrochemical methods, membrane filtering, adsorption in activated carbon, biodegradation, and biosorption. Their main characteristics are described below.

1. **Advanced oxidation processes** allow for pharmaceutical drugs to be degraded using strong oxidant agents such as ozone (O_3_), hydroxyl radicals (-OH^−1^), and hydrogen peroxide (H_2_O_2_); or a combination of compounds such as titanium oxide (TiO_2_) with UV light (photocatalysis); or iron salts and H_2_O_2_ (Fenton) among others [169,170,171,172].
2. **Ionic exchange resins**: Pharmaceutical pollutants in water are removed through electrostatic interaction between charged functional groups in the resin and those in the drug structure. Mineral (clays/zeolite), organic (peat/lignite), and synthetic materials (acrylic acid polymers/Sephadex) are commonly used in these processes [79,173,174].
3. **Chemical precipitation**: The addition of adequate chemicals/additives such as alum, marine salt, or metallic salts (e.g., FeCl_3_, AlCl_3_, MgCl_2_, CaCl_2_) to drug-polluted water generates insoluble chemical complexes/particles that precipitate as sludge, facilitating their removal from the water solution [175,176].
4. **Electrochemical methods**, in which the application of electric current generates reactive species that allow for the oxidation of the pollutant drugs present in the water, a process that leads to degradation [177,178,179].
5. **Membrane filtering**: These drug removal methods use different membranes that effectively exclude pollutants from water based on their molecular size and weight. Reverse osmosis, nanofiltration, and ultrafiltration are the main technologies based on membrane filtering [180,181,182].
6. **Adsorption in activated carbon**: Activated carbon can attract and bind drug molecules to its surface (adsorption), allowing for their effective removal from water [183,184,185,186].
7. **Biodegradation**: Pharmaceutical pollutant degradation is mediated by the metabolic activities of different organisms such as bacteria, fungi, and algae that break drugs down into more straightforward and less harmful substances [187,188,189,190].
8. **Biosorption** is an approach for pharmaceutical pollutant removal from water employing sorbents of biological origin (biomass); these include lignocellulosic materials, such as agro-industrial wastes, woody biomass, or vegetable peels, but also other biological origin materials, including algae, manure, mussel and crustacean shells, or bird feathers. In these materials, pollutants are passively bound through ionic, chemical, or physical mechanisms [191,192,193].

Each method has its own advantages and limitations, and the choice of remediation technique depends on factors such as the specific pharmaceutical compound, its concentration, the water source characteristics, and the desired treatment goals. Integrating multiple treatment methods in a treatment train approach is often necessary to effectively remove pharmaceutical residues from water sources.

### 6.1. Pharmaceutical Removal from Water through Biosorption Using Agro-Industrial Wastes

Due to its low cost and efficiency, biosorption through different materials has taken on great relevance in removing contaminants from water, including heavy metals, hydrocarbons, industrial chemicals, pesticides, and drugs. As materials for the biosorption of pollutants, agro-industrial waste stands out. These materials can be used directly in absorption processes or be subjected to physical, chemical, or thermal treatments to improve their characteristics and efficiency in removing contaminants [192,194,195,196].

Recently, research on the use of biochar in drug removal has taken on great relevance. Biochar is a carbonaceous material produced through the pyrolytic treatment of different biomaterials, including manure generated in livestock, sewage sludge, food waste, and several agro-industrial waste types produced from agricultural practices. Due to its physical, structural, and chemical characteristics, it can efficiently retain large quantities of contaminants [197,198,199,200]. In the present review, studies on removing contaminants of pharmaceutical origin from water through agro-industrial wastes and their conversion into biochar are analyzed.

This review encompasses 94 investigations conducted between 2012 and 2024. Given the diverse experimental conditions in pharmaceutical removal studies, it is challenging to compare the removal capacities of different agro-industrial wastes. To address this, we propose a comparison using the drug mass removal rate (DMRR). Table 1 presents 41 investigations on using agro-industrial waste as biosorbent materials to remove 17 antibiotic molecules from water, covering the period from 2012 to 2024. Among the antibiotic molecules evaluated, sulfamethoxazole was the principal with twelve studies (23.5%), followed by ciprofloxacin with eight studies (15.7%), tetracycline with six studies (11.8%), and amoxicillin with five studies (9.8%). The remotion of antibiotics, norfloxacin, and ofloxacin were evaluated in three studies each (5.9%). At the same time, the removal of chloramphenicol, doxycycline, and metronidazole was assessed in two studies for each antibiotic (3.9%). Finally, removal of the antibiotics azithromycin, cefazolin, sulfadiazine, sulfamethazine, enrofloxacin, trimethoprim, cephalexin, and penicillin G was reported in just one study each (2.0%). It is worth mentioning that in some of the studies presented in Table 1, the removal of more than one antibiotic using the same agro-industrial residue was reported.

Likewise, Table 1 shows the different agro-industrial wastes used as biosorbents in antibiotic removal in water. These materials included wheat straw, walnut shells, bagasse, bamboo, pine waste, alfalfa, grass, peach stones, olive stones, olive pomace, pomegranate peel, rice straw, corn cob, coffee husk, mango seeds, red mombin seeds, cassava starch, vine wood, spent black tea leaves, and bird feathers. In this set of studies, the biosorbent material most used was the activated nanocarbon from vine wood reported by Pouretedal and Sadegh (2014) [201], used for the removal of antibiotics such as amoxicillin, tetracycline, cephalexin, and penicillin G, as well as the activated carbon generated from mango seeds reported by Bednárek et al. (2022) [202] for the removal of norfloxacin and ofloxacin.

On the other hand, in 71% of the studies included in Table 1, agro-industrial wastes were used as raw materials to produce biochar through pyrolysis at different temperatures. Only in 29% of the antibiotic removal studies used non-treated lignocellulosic fibers from agro-industrial waste. Activation is a thermal or chemical process that improves the adsorptive properties of lignocellulosic materials and biochar. The objective of activation is to increase properties such as the surface area, porosity, hydrophobicity, and bulk density of the original raw material. These structural changes increase the removal efficiency of different pollutants [203,204,205]. For the chemical activation of the lignocellulosic fibers and biochar, different compounds were utilized, among them ammonium chloride (NH_4_Cl), zinc chloride (ZnCl_2_), citric acid (C_6_H_8_O_7_), phosphoric acid (H_3_PO_4_), potassium carbonate (K_2_CO_3_), potassium hydroxide (KOH), and sodium hydroxide (NaOH).

The five agro-industrial wastes that showed the highest DMRR values were almond shell-activated biochar (1940 mg/g·h) [206], and biochar of malt rootlets (1740.7 mg/g·h) [207] in the removal of sulfamethoxazole; *Bertholletia excelsa* capsules (920.3 mg/g·h) in the removal of amoxicillin [208]; *Dialium guineense* seed waste sodium hydroxide in modified form (NH-DGS) (762.5 mg/g·h) in the removal of ciprofloxacin [209]; and spent black tea leaves (SBTL) (712 mg/g·h) in the removal of doxycycline [210].

**Table 1:** Removal of antibiotics with agro-industrial wastes.

Pharmaceutical Drug	Chemical Structure	Ci (mg/L)	Agro-Industrial Waste	Agro-Industrial Waste Treatment	Biosorbent Concentration (g/L)	Time (h)	Removal Percentage	q_max_ (mg/g) Langmuir Model	Reference	DMRR (mg/g·h)
Sulfamethoxazole	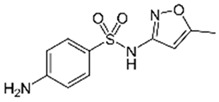	1	*Miscanthus x giganteus* biochar	Air-dried, cut, pyrolyzed at 360 °C.	2.0	4	32	-	[211]	0.3
		3.8	Rice husk	Rice husks were washed, dried at 60 °C for 48 h, grounded, sieved and pyrolyzed at 500 °C (RH-500) and 800 °C (RH-800) for 2 h. RH-800 was activated with NaOH, heated at 800 °C.	0.2	0.25	-	-	[212]	-
		10–60	Sugarcane bagasse	Untreated sugarcane bagasse was used as an adsorbent, was washed with ultrapure water, dried at 60 °C for 8 h, ground, and triturated.	0.02	0.083	51	1.43	[213]	-
		20	Wheat straw ashes	The acidification of ashes was performed using 2 M HCl, mixture at 70 °C, filtered, dried at 105 °C.	1	0.5	22	-	[214]	8.8
Sulfamethoxazole	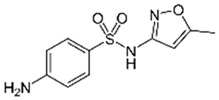	100	Activated biochar of forage bermudagrass	Dried and sieved, pyrolyzed at 300 °C, activated with NaOH, washed with 3 M HCl and deionized water, dried at 105 °C.	0.01	72	91.86	424.66	[215]	127.6
		0.5–40	Activated carbon of walnut shell	Pyrolyzed at 300 °C, impregnated with K_2_CO_3_, heated at 105 °C and 900 °C.	0.01	48	-	93.5	[216]	-
		250	Biochar of malt rootlets	Dried at 50 °C, sieved, heated at 900 °C.	0.09	1.5	94	-	[207]	1740.7
		10	Biochar of bagasse	Pyrolyzed at 300, 400, and 600 °C, washed with deionized water, dried, crushed, and sieved.	0.1	24	41.55	54.38	[217]	1.8
		30	Almond shell activated biochar	Pyrolyzed at 700 °C and mixed with H_2_O_2_.	0.005	3	96.88	344.8	[206]	1940.0
		0.5–50	Bamboo biochar	Cut into 0.6–2 mm size particles, pyrolyzed at 380 °C, cooled, grinded, washed, and dried. Added H_3_PO_4_ at 50 °C, heated to 600 °C, cooled, washed with distilled water, and dryed at 120 °C.	0.1	24	-	34.01	[218]	-
		0.00005	Pelletized pine forestry waste biochar	Pyrolyzed at 850 °C, ground and sieved, dried at 105 °C.	40	1	75	-	[219]	0.0000009
		0–80	*Arundo donax* stems biochar	Pyrolyzed at 300–600 °C, milled, washed with deionized water, and other portion was pulverized for demineralization with HCl and HCL-HF and heated to 750 °C.	0.1	48	-	0.473–0.778 *	[220]	-
Ciprofloxacin	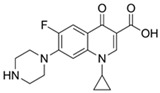	50	Olive stones	Olive stone waste was cleaned with distilled water, dried at 105 °C; was pulverized, washed, and dried at 105 °C; was carbonized at 450 °C; added KOH and activating at 550, 650, and 750 °C.	1	1.25	99	294.98	[221]	39.6
		25	Activated biochar of barley bagasse	The barley bagasse was dried at 60–70 °C, crushed, sieved, and heated at 105 and 400 °C; activated biochar of barley bagasse with 2 M of K_2_CO_3_, dried and washed with distilled water.	0.5	24	82	104.9	[222]	1.7
		5.0	Rice husk	Rice husks were washed, dried at 60 °C for 48 h, grounded, sieved and pyrolyzed 500 °C (RH-500) and 800 °C (RH-800) for 2 h. RH-800 was activated with NaOH, heated at 800 °C.	0.2	0.25	-	584.4	[212]	-
		10–60	Sugarcane bagasse	Untreated sugarcane bagasse was used as an adsorbent, was washed with ultrapure water, dried at 60 °C for 8 h, ground, and triturated.	5	0.083	99	2.61	[213]	-
		250	*Dialium guineense* seed waste (DGS)	Washed with distilled water, dried in open air, heated at 105 °C, dried, grinded, and sieved.	0.1	2	42.9	9.17	[209]	536.0
			*Dialium guineense* seed waste sodium hydroxide modified form (NH-DGS)	Washed with distilled water, dried in open air, heated at 105 °C, dried, grinded, and sieved. Mixed with NaOH, filtered and washed with deionized water, dried at 80–120 °C.			61.0	120.34		762.5
		60	Banyan aerial roots	Cut 1 cm, washed with deionized water, dried at 80 °C, mixed with 1 M C_6_H_8_O_7_, and heated at 150 °C.	0.03	48	90.66	65.70	[223]	37.8
		20	Biochar of rice straw	Washed, dried at 80 °C, crushed, heated to 700 °C, washed with deionized water.	1.6	24	55.0	48.80	[224]	0.3
		100	Carbon from date palm leaflets	Cut, carbonized with sulfuric acid, heated at 160 °C.	0.4	48	-	133.3	[225]	-
Tetracycline	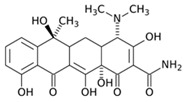	100	Alfalfa biochar	Pyrolyzed at 500 °C, washed with deionized water and filtered (0.45 µm), and washed with 0.1 M HCl.	0.01	48	-	372.31	[226]	-
			Bermudagrass biochar				-	44.24		-
		10	Activated carbon from peach stones (AC-PS)	Washed with ultrapure water, dried at 110 °C, and chemically activated with H_3_PO_4_.	0.8	3	96.1	845.9	[227]	4.0
		100	Activated carbon of hard Shell of apricot stone	Washed with distilled water, ground, added H_3_PO_4_, heated to 100 °C and after to 400 °C, washed with distilled water, dried at 80 °C.	0.3	24	100	308.33	[228]	13.9
		20	Activated nanobiochar from vine wood	Washed, pyrolyzed at 600 °C, activated with NaOH 5% *w*/*w* and NaCl (3 M).	0.4	8	88.17	1.98	[201]	5.5
		100	Spent black tea leaves (SBTL)	Steep under stirring with boiling water 100 °C, filtered, washed with distilled water, dried at 100 °C, ground.	0.2	3	78	-	[229]	130.0
			Pomegranate peel (PP)	Ground and pulverized, steeped under stirring with boiling water 100 °C, filtered, washed with distilled water, dried at 100 °C, ground.	0.3	0.5	90	-		600.0
		50	Bamboo charcoal	Carbonized at 150–250 °C, 250–400 °C and 400–700 °C, ground and sieved, washed, dried at 105 °C.	1	24	87.6	22.7	[230]	1.8
Amoxicillin	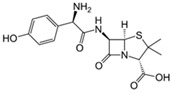	25	Banana peel activated carbon	Activated carbon by H_3_PO_4_, carbonized at 350 °C, 450 and 500 °C.	1.5	2	96.02	393.70	[231]	8.0
		100	Activated biochar from corn cob	Corn cobs were used for activated biochar with ZnCl_2_, dried at 120 °C for 12 h, pyrolyzed at 700 °C for 2 h.	1	6	65.88	175.86	[232]	11.0
		700	*Bertholletia excelsa* capsules	Milled, mixed with ZnCl_2_, dried at 80 °C, pyrolyzed from 25 up to 600 or 700 °C, cooled, and refluxed with HCl.	1.5	0.5	98.60	388.4	[208]	920.3
		25	Olive stone	Washed with tap water and impregnated with H_3_PO_4_ at 100 °C for 9 h, dried at 170 °C and 380 °C, washed with distilled water, and dyed at 110 °C.	1.0	100	93	57.04	[233]	0.2
		20	Activated nanobiochar from vine wood	Washed, pyrolyzed at 600 °C, activated with NaOH 5% *w*/*w* and NaCl (3 M).	0.4	8	60.23	2.69	[201]	3.8
Norfloxacin	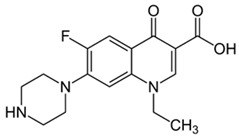	15–150	Activated carbons of red mombin seeds	The red mombin seeds, corn cob, coffee husk, internal and external parts of mango seeds and ice cream beans were washed with potable water and dried at 80 °C, were grounded and sieved, all raw materials were mixed with ZnCl_2_ and were pyrolyzed at 600 °C for 2 h.	0.01	7	-	404	[202]	-
			Activated carbons of corn cob				-	264		-
			Activated carbons of coffee husk				-	235		-
			Activated carbons of internal parts of mango seeds				-	221		-
			Activated carbons of external parts of mango seeds				-	262		-
			Activated carbons of ice cream beans				-	84		-
		5	Rice husk	Washed with distilled water, dried at 60 °C for 48 h, ground to powder and sieved.	0.2	3	59.51	-	[234]	5.0
			Coffee husk				70.83	-		5.8
		10	Biochar from potato stem	Washed, dried at 80 °C, chopped, crushed, sieved, pyrolyzed at 500 °C.	0.1	36	-	5.24	[235]	-
Ofloxacin	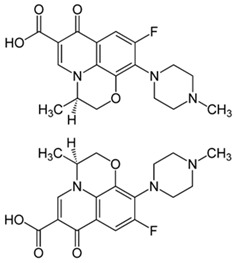	25–250	Activated carbons of red mombin seeds	The red mombin seeds, corn cob, coffee husk, internal and external parts of mango seeds and ice cream beans were washed with potable water and dried at 80 °C, were grounded and sieved, all raw materials were mixed with ZnCl_2_ and were pyrolyzed at 600 °C for 2 h.	0.01	7	-	380	[202]	-
			Activated carbons of corn cob				-	267		-
			Activated carbons of coffee husk				-	202		-
			Activated carbons of internal parts of mango seeds				-	176		-
			Activated carbons of external parts of mango seeds				-	254		-
			Activated carbons of ice cream beans				-	77		-
		100	Bamboo sawdust biochar	Pyrolyzed at 500 °C, washed with distilled water, dried at 105 °C.	0.05	96	-	45.11	[236]	-
		50	*Moringa oleifera* pod husks (AMOP)	Air-dried, pulverized, sieved, activated with NH_4_Cl, filtered, washed with distilled water, and air-dried.	2.5	4	90.98	3.597	[237]	4.5
			Biochar of *Moringa oleifera* pod husks (CMOP)	Air-dried, pulverized, sieved, activated with NH_4_Cl, filtered, washed with distilled water, air-dried, pyrolyzed at 350 °C, washed with distilled water, air-dried.			99.84	5.051		5.0
Chloramphenicol	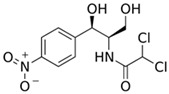	5–100	Wheat straw	Pyrolyzed at 550 °C and 700 °C, respectively, crushed and sieved, washed with HCl and deionized water.	0.05	24	-	11.3	[238]	-
			Softwood				-	8.8		-
		50	Bamboo charcoal	Carbonized at 150–250 °C, 250–400 °C and 400–700 °C, ground and sieved, washed, dried at 105 °C.	1.0	24	70.3	8.1	[230]	1.5
Doxycycline	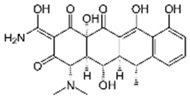	20	Biochar of rice straw	Washed, dried at 80 °C, crushed, heated to 700 °C, washed with deionized water.	1.6	24	90.0	170.36	[224]	0.5
		100	Spent black tea leaves (SBTL)	Steep under stirring with boiling water 100 °C, filtered, washed with distilled water, dried at 100 °C, ground.	0.05	2.5	89	-	[229]	672.0
			Pomegranate peel (PP)	Ground and pulverized steep under stirring with boiling water 100 °C, filtered, washed with distilled water, dried at 100 °C, ground.	0.15	1.5	83	-		368.9
Metronidazole	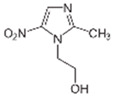	100	Cassava starch waste	Dried at 60 °C, activated with H_3_PO_4_, NaOH and combination of both, heated at 105 °C, washed with water, dried at 105 °C, pyrolyzed at 900 °C.	0.1	24	55	-	[239]	22.9
		0.5–40	Activated carbon of walnut Shell	Pyrolyzed at 300 °C, impregnated with K_2_CO_3_, heated at 105 °C and 900 °C.	0.01	48	-	93.5	[216]	-
Azithromycin	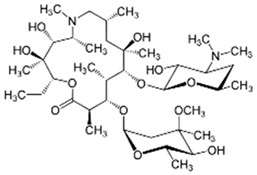	200	Biochar from *Terminalla chebula* (CBC)	Cleaned, dried, and peeled the bagasse was shredded, washed, dried 72 h at 70 °C, heated for pyrolysis at 500 °C for 1 h; after cooling, washed and dried at 70 °C for 12 h.	200	2	57.17	21.36	[240]	0.29
			Biochar from *sugarcane bagasse* (BBC)				60.03	17.95		0.30
Cefazolin	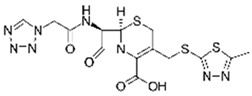	30	Alperujo	Alperujo dried were covered with distilled water, heated at 160–220 °C, dried at 50 °C, and sieved.	0.125	24	-	1572.73	[241]	-
Sulfadiazine	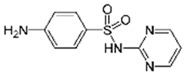	20	Activated carbons from olive pomace	Washed with hot distilled water, dried at 103 °C, ground and sieved, pyrolyzed at 450 °C, impregnated with KOH, stirred at 60 °C, dried at 103 °C. The mixture was activated at 560, 700, and 840 °C, cooled, washed with distilled water, and dried at 103 °C.	0.8	2	99	66.2	[242]	12.3
Sulfamethazine	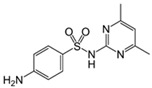	0.5–50	Bamboo biochar	Cut into 0.6–2 mm size particles, pyrolyzed at 380 °C, cooled, grinded, washed, and dried. Adding H_3_PO_4_ at heated 50 °C and heated at 600 °C, cooled, washed with distilled water and drying at 120 °C.	0.1	24	-	40.11	[218]	-
Enrofloxacin	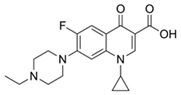	100	Bamboo sawdust biochar	Pyrolyzed at 500 °C, washed with distilled water, dried at 105 °C.	0.05	96	-	45.88	[236]	-
Trimethoprim	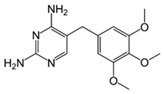	29	Charcoal from feathers	Washed, drying at 80 °C, crushed, heated at 600 °C, dried 105 °C, sieved to 100–160 mesh.	0.2	60	90	164	[243]	2.2
Cephalexin	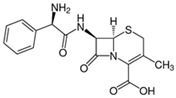	20	Activated nanobiochar from vine wood	Washed, pyrolyzed at 600 °C and activated with NaOH 5% *w*/*w* and NaCl (3 M).	0.4	8	76.02	7.08	[201]	4.8
Penicillin G	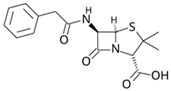	20	Activated nanobiochar from vine wood	Washed, pyrolyzed at 600 °C and activated with NaOH 5% *w*/*w* and NaCl (3 M).	0.4	8	73.94	8.41	[201]	4.6

Ci = initial concentration; q_max_ = maximum absorption according to the adsorption isotherm model Langmuir/Freundlich; DMRR = drug mass removal rate, calculated in this review; * adsorption isotherm model: Freundlich.

Concerning the removal of anti-inflammatory drugs from water, 36 studies were identified on the evaluation of six non-steroidal anti-inflammatory drugs (NSAIDs) and their removal through agro-industrial wastes between 2012 and 2024 (Table 2). The NSAIDs included in the studies were diclofenac with 16 studies (36.4%), acetaminophen with 13 studies (29.5%), ibuprofen with 12 studies (27.3%), and dipyrone, ketoprofen, and naproxen with 1 study each (2.3%). These NSAID molecules were removed using 41 different agro-industrial wastes, among them mung bean husk, cocoa pod husks, potato peel waste, *Quercus brantii* (Oak), residual pods of *Erythrina speciose*, rice husk, banana leaves, Jatoba barks, olive stones, olive waste cake, onion skin, bean husks, cellulose fiber, fique Bagasse, Moringa oleifera seeds husks, pinewood biochar, softwood, wheat straw, wheat straw ashes, wood apple biochar, and wood apple stems, among other wastes. Some agro-industrial wastes were employed in the removal of different NSAIDs. For example, in the study of Baccar et al. (2012) [244], activated biochar from olive waste cake was evaluated in the removal of diclofenac, ibuprofen, ketoprofen, and naproxen.

Of the 41 agro-industrial wastes used in the removal of NSAIDs, 25 (61%) were transformed biochar. In comparison, lignocellulosic fibers without treatment of three agro-industrial wastes were employed in the studies of NSAIDs removal. Likewise, biochar and lignocellulosic fibers were activated using different chemical reagents, and phosphoric acid (H_3_PO_4_) was used to activate the following residues: hydrochloric acid (HCl), zinc chloride (ZnCl_2_), citric acid (C_6_H_8_O_7_), potassium hydroxide (KOH), ammonium chloride (NH_4_Cl), carbon dioxide (CO_2_), sodium hydroxide (NaOH), sulfuric acid (H_2_SO_4_), iron(III) chloride (FeCl_3_), iron(II) sulfate (FeSO_4_), nitric acid (HNO_3_), and, finally, potassium carbonate (K_2_CO_3_).

According to these results, the five agro-industrial wastes that showed the highest DMMR values were cork powder (CP) (10,420 mg/g·h), old coffee grounds (CG) (9400 mg/g·h), yeast waste (YW) (6200 mg/g·h), [245], *Gundelia tournefortii* seeds (786.5 mg/g·h) [246], and activated carbon from cocoa pod husks (449.3 mg/g·h) [247].

The removal of other groups of drug pollutants other than antibiotics and NSAIDs from water was also evaluated. Table 3 includes 26 studies on the use of agro-industrial waste in the removal of 27 different pharmaceutical molecules; the most evaluated drug molecules include carbamazepine (antiepileptic), assessed in eight studies (20%), and triclosan (antibacterial and fungicide), assessed in three studies (7%). The studies evaluate the removal of 25 additional drug molecules that include 6 additional neurological/psychoactive drugs (diazepam, fluoxetine, oxazepam, paroxetine, pramipexole, and sertraline), 6 additional antimicrobial/antiparasitic drugs (albendazole, chloroquine, sulfapyridine, and sulfathiazole), sex hormones (17α-ethinyl estradiol, 17β-estradiol, estriol, and estrone), and glucocorticoids (dexamethasone), among other pharmaceutical pollutants such as anticancerogenics, anticholesterolemics, antidiabetics, antiemetics, antihistamines, antihypertensives, cosmetic sunlight protectors, keratolytic agents, and ophthalmic medications.

**Table 2:** Removal of non-steroidal anti-inflammatory drugs (NSAIDs) with agro-industrial wastes.

Pharmaceutical Drug	Chemical Structure	Ci (mg/L)	Agro-Industrial Waste	Agro-Industrial Waste Treatment	Biosorbent Concentration (g/L)	Time (h)	Removal Percentage	q_max_ (mg/g) Langmuir Model	Reference	DMRR (mg/g·h)
Diclofenac	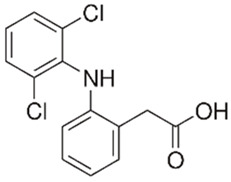	50	Olive stones	Olive stone waste was cleaned with distilled water; dried at 105 °C; pulverized, washed, and dried at 105 °C; carbonized at 450 °C; and KOH was added and activated at 550, 650, and 750 °C.	1	1.25	99	256.41	[221]	39.6
		25	Chestnut shells biochar	The chestnut shells were dried at 60 °C for 8 h and ground; 50 g of water chestnut shells were soaked in FeSO_4_.7H_2_O, pyrolyzed at 600 °C, sieved, and dried at 80 °C.	0.5	1	98	75.9	[248]	49.0
		4.4	Rice husk	Rice husks were washed, dried at 60 °C for 48 h, grounded, sieved, and pyrolyzed 500 °C (RH-500) and 800 °C (RH-800) for 2 h. RH-800 was activated with NaOH, heated at 800 °C.	0.2	0.25	-	-	[212]	-
		100	Rice husk ash	Rice husk ashes were heated at 105 °C for 1.5 h and sieved.	1	4	-	2.3	[249]	-
		10–60	Sugarcane bagasse	Untreated sugarcane bagasse was used as an adsorbent, washed with ultrapure water, dried at 60 °C for 8 h, ground, and triturated.	0.017	0.083	77	1.81	[213]	-
		5–100	Wheat straw	Pyrolyzed at 550 °C and 700 °C, respectively, crushed and sieved, washed with HCl and deionized water.	0.05	24	-	6.7	[238]	-
			Softwood				-	5.5		-
		12.5	Fique Bagasse	Dried at 100 °C, sieved, and biochar produced through pyrolyzed at 650, 750, and 850 °C.	0.05	24	56	5.4	[250]	5.8
		20	Wheat straw ashes	The acidification of ashes was carried out using 2 M HCl, mixture at 70 °C, filtered, dried at 105 °C.	1	0.5	0	-	[214]	0.0
		0.5	Pinewood microbiochar	Pyrolyzed at 525 °C, ground and sieved.	2	48	70	1.106 **	[251]	0.004
		20–350	*Moringa oleifera* seed husks	Husks were peeled and ground, washed, dried at 105 °C, mixed with methyl alcohol, washed with purified water and stirred in HNO_3_, washed with purified water and dried at 105 °C, dried at 300 °C, washed with purified water, filtered, and dried at 105 °C.	0.025	24	-	72.77	[252]	-
		0.5	Pinewood microbiochar	Pyrolyzed at 525 °C, ground, and sieved.	0.1	4.5	68	526.3	[253]	0.8
		10	Activated onion skin	Washed, dried and ground (0.25 mm), pretreated with H_2_SO_4_, and dried at 50 °C.	0.05	3.6	65.99	134.0035 *	[254]	36.7
		30	Activated carbon from cocoa pod husks	Sun-dried cocoa pods were pulverized, the powder was sieved, soaked for 24 h with H_2_SO_4_, washed with deionized water, and dried at 120 °C.	0.25	0.25	93.6	0.47	[247]	449.3
		0.1	Cellulose fiber (CF) decorated with polypyrrole (PPy)	CF mixed with FeCl_3_.6H_2_O, filtered, washed with water/methanol (1:1, *v*/*v*), dried at 60 °C.	0.025	0.25	93	210.07	[255]	14.9
		50	Activated carbon from potato peel waste	Dried at 60 °C, milled and sieved, carbon activation was carried out with K_2_CO_3_, dried at 100 °C, heated at 700 °C, cooled, washed with distilled water, and dried at 100 °C.	0.01	24	70	68.5	[256]	145.8
		14.80	Activated carbon of olive waste cake	Impregnated with H_3_PO_4_, pyrolyzed to 450 °C, cooled, washed with hot distilled water, dried at 105 °C, and ground.	0.3	26	-	56.2	[244]	-
Acetaminophen	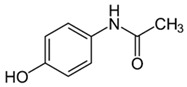	25–100	Tingui shells	Tingui shells underwent thermal decomposition at 550 °C for 2 h and activation with carbon dioxide, and distilled water was added; it was kept at 200 °C for 24 h and activated with carbon dioxide; called HT-CO_2_.	20	24	-	383.2	[257]	-
		50	Activated carbon from artichoke leaves	Pomegranate peels and artichoke leaves were washed with deionized water and dried for 36 h at 90 °C, crushed and sieved, and dried at 115 °C for 24 h. The pyrolysis was carried out at 450 °C and peels were mixed with 1 M HCl at 80 °C for 1.5 h.	0.5	1	98.1	154.9	[258]	49.1
			Activated carbon from pomegranate peels				-	258.9		-
		2.3	Rice husk	Rice husks were washed, dried at 60 °C for 48 h, grounded, sieved, and pyrolyzed at 500 °C (RH-500) and 800 °C (RH-800) for 2 h. RH-800 was activated with NaOH, heated at 800 °C.	0.2	0.25	-	209.6	[212]	-
Acetaminophen	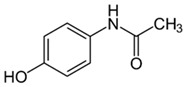	12.5–200	Purple basil (*Ocimum basilicum* L.) waste	The plant was collected, washed, dried for 5 days, pulverized, mixed with distilled water, heated at 85 °C for 60 min, cooled, and filtered.	0.5	1–800	-	0.023	[259]	-
		100	Activated biochar from corn cob	Corn cobs were used for activated biochar with ZnCl_2_, dried at 120 °C for 12 h, and pyrolyzed at 700 °C for 2 h.	1	6	69.37	332.08	[232]	11.6
		100	Dende coconuts (DND)	The carbons derived from babassu coconut biomass and dende coconut were sieved, washed with water, and dried at 60 °C for 24 h.	10	300	-	149	[260]	-
			babassu coconuts (BBS)				-	128		-
		100	*Gundelia tournefortii* seeds	*Gundelia tournefortii* seeds were washed with distilled water and dried. After, the seed waste was soaked for 24 h in H_3_PO_4_, washed with distilled water, dried at 105 °C for 24 h, and then carbonized for 1 h at 700 °C, cooled, washed, and dried at 105 °C.	0.25	0.5	98.31	14.34	[246]	786.5
		150	Mango seeds	Mango seeds were cut, rinsed in distilled water, drained, and immersed in HCl for 24 h. After, mango seeds were washed with distilled water and dried at 105 °C; crushed, sieved, and treated with phosphoric acid (H_3_PO_4_) for 24 h; dried at 105 °C for 24 h; and carbonized at 400 °C for 1 h. Rinsed with NaOH and dried at 105 °C.	1.95	1.1	94.01	7.23	[261]	65.7
		45	Activated carbon from residual pods of *Erythrina speciosa*	Washed with tap water, broken, dried at 50 °C, grounded, mixed with ZnCl_2_, dried, heated at 10 °C, pyrolyzed at 800 °C, washed with HCl and distilled water, dried at 50 °C.	1.2	2	86.49	60.83	[262]	16.2
		450	Activated carbon of Jatoba bark	Washed with distilled water, dried at 70 °C, ground, activated with KOH, heated at 500 °C.	0.025	4	-	356.25	[263]	-
		100	Activated carbon from *Quercus brantii* (Oak)	Pyrolysis at 600 °C and activation with NaOH, KOH, NH_4_Cl, ZnCl_2_, and H_3_PO_4_.	10	2.5	89.55	45.45	[264]	3.6
		120	Activated carbon of banana leaves	Cleaned with tap water, washed, dried, crushed, and immersed in H_2_SO_4_; carbonized at 150 °C, cooled, washed with NaOH and distilled water, and dried at 60 °C.	0.01	24	83.8	142.2	[265]	419.2
		0.007	Activated carbon of olive stones	Grinded, washed with distilled water, dried at 100 °C, impregnated with H_3_PO_4_, heated at 500 °C, washed with distilled water at 60 °C, heated at 900 °C, grinded, and sieved.	0.01	240	-	108.3	[266]	-
Ibuprofen	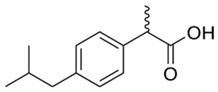	100	Rice husk ash	Rice husk ashes were heated at 105 °C for 1.5 h and sieved.	1	4	-	2.3	[249]	-
		10–60	Sugarcane bagasse	Untreated sugarcane bagasse was used as an adsorbent; washed with ultrapure water, dried at 60 °C for 8 h, ground, and triturated.	0.02	0.083	65	1.62	[213]	-
		10	Olive pomace	The olive pomace was dried at 105 °C for 24 h and sieved, activated with zinc chloride and calcium hydroxide (1:0.8:0.2), and pyrolyzed at 550 °C for 0.5 h; after, the treated material underwent acid leaching with HCl 6 mol L^−1^ and was washed with water, dried at 105 °C for 24 h.	0.5	0.8	95.28	360.607	[267]	2.8
		75	Activated carbon from rose geranium (*Pelargonium graveolens* L.) leaves	The rose geranium (*Pelargonium graveolens* L.) leaves were pulverized, pyrolyzed at 600 °C for 2 h; the activation of carbon leaves was with orthophosphoric acid (H_3_PO_4_); after, the mixture was rinsed with ultrapure water several times and dried at 70 °C.	0.5	1.5	83.12	113.76	[268]	83.1
		50	Carbon activated of bean husks	Washed with distilled water, sun-dried, pulverized, sieved H_3_PO_4_ added, heated at 105 °C, cooled and washed with deionized water, and dried at 105 °C.	0.1	1.16	78.17	50.00	[269]	336.9
		15	Wood apple biochar (WAB)	Pyrolyzed at 650 °C, heated to 65 °C, and cooled.	0.33	24	90	5	[270]	1.7
		30	Wood apple steam activated biochar (WASAB)		1		95	12.658		1.2
		100	Activated carbon from *Quercus brantii* (Oak)	Pyrolysis at 600 °C and activation with NaOH, KOH, NH_4_Cl, ZnCl_2_, and H_3_PO_4_.	10	2	100	96.15	[264]	5.0
		10	Activated onion skin	Washed, dried and ground (0.25 mm), pretreated with H_2_SO_4_, and dried at 50 °C.	0.05	3.6	81.90	147.058	[254]	45.6
		10	Activated carbon from rice husk (AC-RH)	Washed with ultrapure water, dried at 110 °C, and chemically activated with H_3_PO_4_.	0.8	3	97.2	239.8	[227]	4.0
		20	Activated biochar from mung bean husk	Washed with distilled water, dried, pyrolyzed at 550 °C, and heated up to 650 °C; steam was passed for activate biochar; was grinded and sieved.	0.1	2	99.16	62.5	[271]	99.2
		100	Pinewood biochar	Pyrolyzed at 425 °C, washed with distilled water, sieved, heated at 110 °C.	4	16	72.0	10.74	[272]	1.2
		10.04	Activated carbon of olive waste cake	Impregnated with H_3_PO_4_, pyrolyzed to 450 °C, cooled, washed with hot distilled water, dried at 105 °C, and ground.	0.3	26	70.07	12.6	[244]	0.9
Dipyrone	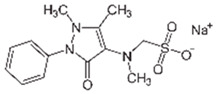	100	Yeast waste (YW)	The wastes YW, CP, and CG were pyrolyzed at 800 °C for 2 h. For functionalization, natural deep eutectic solvent (NADES) was added to the biochar, also mixing citric acid, sucrose, and water (1:1:10); was stirred at 220 rpm for 2 h at 50 °C. The suspensions were ultrasonicated at 65 °C for 2 h and dried at 65 °C.	0.01	0.5	31	2.71	[245]	6200
			Cork Powder (CP)			0.5	52.10	14.66		10,420
			Old coffee grounds (CG)			0.5	47	40.78		9400
Ketoprofen	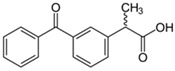	19.28	Activated carbon of olive waste cake	Impregnated with H_3_PO_4_, pyrolyzed to 450 °C, cooled, washed with hot distilled water, dried at 105 °C, and ground.	0.3	26	88.40	24.7	[244]	2.2
Naproxen	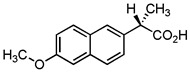	19.78	Activated carbon of olive waste cake	Impregnated with H_3_PO_4_, pyrolyzed to 450 °C, cooled, washed with hot distilled water, dried at 105 °C, and ground.	0.3	26	90.45	39.5	[244]	2.3

Ci = initial concentration; q_max_ = maximum absorption according to the adsorption isotherm model Langmuir/Freundlich; DMRR= drug mass removal rate, calculated in this review; * adsorption isotherm model: Freundlich; ** adsorption isotherm model: Thomas.

**Table 3:** Removal of diverse groups of pharmaceutical pollutants with agro-industrial wastes.

Pharmaceutical Drug	Chemical Structure	Ci (mg/L)	Agro-Industrial Waste	Agro-Industrial Waste Treatment	Biosorbent Concentration (g/L)	Time (h)	Removal Percentage	q_max_ (mg/g) Langmuir Model	Reference	DMRR (mg/g·h)
**Neurological/Psychoactive**										
Carbamazepine ^1^	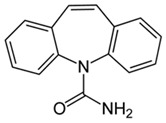	25	Banana peel activated carbon	Activated carbon by H_3_PO_4_, carbonized at 350 °C, 450 °C, and 500 °C.	1.5	2	90.62	338.98	[231]	7.6
		5–100	Wheat straw	Pyrolyzed at 550 °C and 700 °C, respectively, crushed and sieved, washed with HCl and deionized water.	0.05	24	-	15.9	[238]	-
			Softwood				-	20.5		-
		4.7	Biochar of paper mill sludge	Pyrolyzed at 800 °C, washed with HCl and distilled water, and dried at 105 °C.	0.15	0.25	-	17.48	[273]	-
		100	Activated biochar from grapefruit peel	Pyrolyzed at 400 °C, mixed with KOH.	0.01	24	58.6	286.5	[274]	244.2
		0.005	Pinewood nanobiochar	Pyrolyzed at 525 °C; nanobiochar with an average size of 60 ± 20 nm.	0.005	3	95	0.074	[275]	0.3
		100	Activated carbons from peach stones	Crushed and sieved, added H_3_PO_4_, calcined at 435 °C, washed with ultrapure water, and dried at 110 °C.	0.12	4	-	335	[276]	-
		5	Rice straw	Dried, cut, pulverized, sieved, and dried at 60 °C.	60	2	75.3	40.0	[277]	1.2
Fluoxetine ^2^	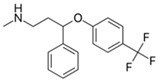	30	Alperujo	Dried alperujo was covered with distilled water, heated at 160–220 °C, dried at 50 °C, and sieved.	0.125	24	-	33.24	[241]	-
Paroxetine ^2^	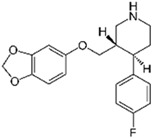	6.6	Biochar of paper mill sludge	Pyrolyzed at 800 °C, washed with HCl and distilled water, and dried at 105 °C.	0.15	0.25	-	21.08	[273]	-
Sertraline ^2^	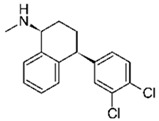	20	Mango stones	Stones were washed with deionized water and dried at 105 °C for 24 h, ground, sieved, and FeSO_4_.7H_2_O was added; the biosorbent was filtered, washed, and dried at 105 °C.	0.01	24	76.97	64.79	[278]	64.1
Diazepam ^3^	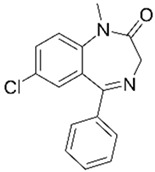	0.00034	Bagasse	Biochar of bagasse was pelletized, and two natural draft gasifier stoves (N1 and N2) were used.	0.05	24	78	-	[279]	0.00022
Oxazepam ^3^	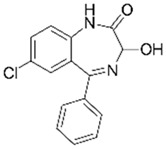	5.7	Biochar of paper mill sludge	Pyrolyzed at 800 °C, washed with HCl and distilled water, and dried at 105 °C.	0.15	0.25	-	20.07	[273]	-
Pramipexole ^4^	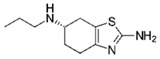	50	Activated carbon from potato peel	Washed with distilled water, dried 120 °C, heated to 200 °C, mixed with KOH, filtered, dried at 100 °C, and activated at 400, 600, and 800 °C.	1	24	61	98	[280]	1.3
**Antimicrobials/Antiparasitics**										
Triclosan	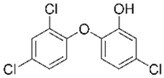	3–60	Wheat straw	Pyrolyzed at 550 °C and 700 °C, respectively, crushed and sieved, washed with HCl and deionized water.	0.05	24	-	20.3	[238]	-
			Softwood				-	30.2		-
		20	Wheat straw ashes	The acidification of ashes was carried out using 2 M HCl, mixture at 70 °C, filtered, dried at 105 °C.	1	0.5	30	-	[214]	12.2
Chloroquine	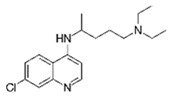	20	Mango stones	Stones were washed with deionized water and dried at 105 °C for 24 h, ground, sieved, and added with FeSO_4_.7H_2_O; the biosorbent was filtered, washed, and dried at 105 °C.	0.01	24	95.95	49.92	[278]	80.0
		100	Sugarcane bagasse	Sugarcane bagasse was dried 24 h at 100 °C, and then was milled, sieved, and stored in a freezer at −2 °C until being modified as hydrochar.	0.02	1	63.69	73.45	[281]	3184.5
Albendazole	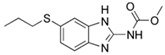	75	Activated carbons of acorns of cork	Pyrolyzed at 500 °C, impregnated with H_3_PO_4_.	2.4	1	99.97	137.2	[282]	31.2
Sulfapyridine	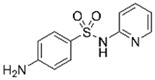	10	Biochar of bagasse	Pyrolyzed at 300, 400, and 600 °C; washed with deionized water; dried; crushed; and sieved.	0.1	24	69.64	8.60	[217]	2.9
Sulfathiazole	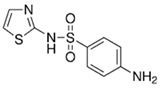	0.5–50	Bamboo biochar	Cut into 0.6–2 mm size particles, pyrolyzed at 380 °C, cooled, ground, washed, and dried. Adding with H_3_PO_4_, heated 50 °C and heated at 600 °C, cooled, washed with distilled water, and dried at 120 °C.	0.1	24	-	25.11	[218]	-
**Hormones**										
17α-ethinyl estradiol	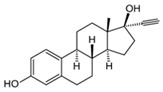	0.2632	Raw peanut shells (*Arachis hypogaea*)	Raw peanut shells underwent washing, drying, grinding, sieving, and chemical treatments.	2	24	90	0.0173	[283]	0.0049
		20	Wheat straw ashes	The acidification of ashes was accomplished using 2 M HCl, mixture at 70 °C, filtering, drying at 105 °C.	1	0.5	15	-	[214]	6.0
17β-estradiol	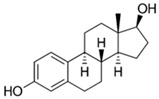	5	Rice husk	The biochar of rice husks was obtained by pyrolysis at 600 °C.	0.5	1	60.1	-	[284]	6.0
		11.3	Rice husk	Washed with ultrapure water, agitated to 200 rpm for 24 h, and filtered in cellulose ester membranes (0.45 µm).	12	1	94.9	1.649	[285]	0.4
Estriol	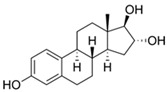	8.0	Rice husk	Washed with ultrapure water, agitated to 200 rpm for 24 h, and filtered in cellulose ester membranes (0.45 µm).	12	1.5	82.5	0.979	[285]	0.3
Estrone	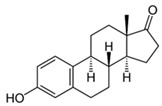	5	Rice husk	The biochar of rice husks was obtained by pyrolysis at 600 °C.	0.5	1	44.6	-	[284]	4.5
		10.5	Rice husk	Washed with ultrapure water, agitated to 200 rpm for 24 h, and filtered in cellulose ester membranes (0.45 µm).	4	0.5	86.3	2.698	[285]	2.3
Dexamethasone ^5^	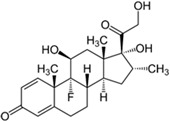	4	Activated biochar of *Syzygium cumini* leaves	Washed with potable water, dried at 60 °C, ground, sieved, mixed with ZnCl_2_, and pyrolyzed at 600 °C.	5	3	53.02	0.673	[286]	0.1
**Other**										
Octocrylene ^6^	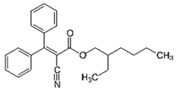	5	Rice husk	The biochar of rice husks was obtained by pyrolysis at 600 °C.	0.5	1	59.4	-	[284]	5.9
Oxybenzone ^6^	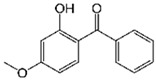	5	Rice husk	The biochar of rice husks was obtained by pyrolysis at 600 °C.	0.5	1	37.4	-	[284]	3.7
Salicylic acid ^7^	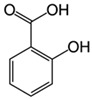	100	Pinewood biochar	Pyrolyzed at 425 °C, washed with distilled water, sieved, heated at 110 °C.	4	16	76.0	22.70	[272]	1.2
Atenolol ^8^	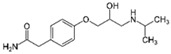	1	*Miscanthus x giganteus* biochar	Air-dried, cut, pyrolyzed at 360 °C.	0.5	4	69	-	[211]	0.3
Carboplatin ^9^	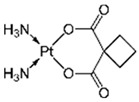	100	Rice husk ash	Rice husk ashes were heated at 105 °C for 1.5 h and sieved.	1	1	36.52	9.9	[249]	36.5
Clofibric acid ^10^	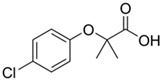	5	Rice straw	Dried, cut, pulverized, sieved, and dried at 60 °C.	30	2	42.5	126.3	[277]	0.04
Dorzolamide ^11^	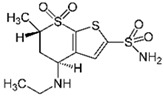	50	Activated carbon from potato peel	Washed with distilled water, dried at 120 °C, heated at 200 °C, mixed with KOH, filtered, dried at 100 °C, and activated at 400, 600, and 800 °C.	1	24	55	92	[280]	1.1
Metformin ^12^	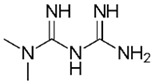	30	Seed husks of *Moringa oleifera* Lam.	*Moringa oleifera* Lam. husks were peeled and washed with deionized water at 45 °C, transferred to nitric acid for 1 h, dried for 12 h, placed in an oven at 300 °C for 1 h, cooled, ground, and sieved.	0.03	15	93.54	28.05	[287]	62.4
Metoclopramide ^13^	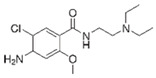	0.000299	Bagasse	Biochar of bagasse was pelletized and used two natural draft gasifier stoves (N1 and N2).	0.05	24	97	-	[279]	0.00024
Ranitidine ^14^	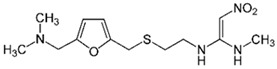	100	Activated carbon of mung bean husk (MBH)	Washed with distilled water, dried, carbonized at 550 °C.	0.75	1.5	99.16	28	[288]	88.1

Ci = initial concentration; q_max_ = maximum absorption according to the adsorption isotherm model Langmuir/Freundlich; DMRR= drug mass removal rate, calculated in this review; ^1^ antiepileptic; ^2^ antidepressant; ^3^ antianxiety; ^4^ dopaminergic; ^5^ glucocorticoid, ^6^ cosmetic sunlight protector; ^7^ keratolytic agent; ^8^ antihypertensive; ^9^ anticancerogenic; ^10^ anticholesterolemic; ^11^ ophthalmic; ^12^ antidiabetic; ^13^ antiemetic; ^14^ antihistaminic.

In the removal of the previously mentioned pharmaceutical drugs, 25 biosorbent materials derived from agro-industrial wastes were used; these included acorns of cork, alperujo, bagasse, bamboo, banana peel, grapefruit peel, mango stones, *Miscanthus x giganteus*, mung bean husk, paper mill sludge, peach stones, peanut shells, pinewood, potato peel, rice husk, rice straw, seed husks of *Moringa oleifera*, softwood, sugarcane bagasse, *Syzygium cumini* leaves, and wheat straw.

Of these 25 agro-industrial wastes evaluated, thirteen were converted into biochar (52%) and nine agro-industrial wastes were used without treatment (36%); among them, bagasse, rice husk, rice straw, and wheat straw were the most representative. Biochar chemical activation was a frequent method to improve removal of pharmaceutical drugs. The main chemical compounds used in biochar and fiber activation were hydrochloric acid (HCl), phosphoric acid (H_3_PO_4_), potassium hydroxide (KOH), and zinc chloride (ZnCl_2_).

Among the agro-industrial wastes, five showed particularly high DMMR values. These were sugarcane bagasse (3184.5 mg/g·h) [281] and mango stones (80 mg/g·h) [278] in the removal of chloroquine; activated biochar from grapefruit peel (244.2 mg/g·h) in the removal of carbamazepine [274]; mango stones (64.1 mg/g·h) in the removal of sertraline [278]; seed husks of *Moringa oleifera* (62.4 mg/g·h) in the removal of metformin [287]; and rice husk ash in the removal of carboplatin [249]. These results underscore the effectiveness of these agro-industrial wastes in removing pharmaceutical pollutants from water.

Overall, the agro-industrial wastes that showed a higher DMRR for each group of pharmaceutical pollutants were almond shell-activated biochar (1940 mg/g·h) for antibiotics, cork powder (10,420 mg/g·h) for NSAIDs, sugarcane bagasse (3184.5 mg/g·h) for chloroquine, and activated biochar from grapefruit peel (244.2 mg/g·h) used in the removal of carbamazepine. Most of these materials could be generated and were highly available only in specific areas, thus limiting their use in the removal of pharmaceuticals on a broader scale. Agro-industrial wastes derived from crops with higher global distribution (e.g., corn, rice, soy, sugarcane, wheat) as raw materials for the removal of pharmaceutical molecules from water could be a better option if their removal efficiency is comparable to that of the above-mentioned materials.

Moreover, all the studies included in this review were conducted in batch systems to evaluate the removal of single pharmaceutical molecules; the main differences among the analyzed studies were the type of drug studied, the initial concentration of the drug, the concentration of the biosorbent material used in the system, and the contact time used to evaluate the removal. Future trends in the evaluation of agro-industrial wastes for the removal of pharmaceutical pollutants from water could include removal studies in continuous systems and the treatment of mixtures of pharmaceutical pollutants. Research approaches could be closer to the reality of the problem, since in aquatic ecosystems with presence of pharmaceutical pollutants, mixtures of multiple molecules with different pharmacological and ecotoxicological adverse effects can be identified.

### 6.2. Management and Treatment Alternatives for Biosorbent Materials Employed for Pharmaceutical Pollutants Removal from Water

At the end of the process, the agro-industrial materials used to remove pharmaceutical pollutants from water could be considered hazardous. Due to this, adequate management and treatment are needed to avoid secondary contamination and ensure environmental safety and sustainability. Different feasible alternatives have been proposed for adequate management and treatment of the agro-industrial wastes used in the treatment of water with the presence of pharmaceutical pollutants, among them, their contention in landfills, incineration, detoxification through chemical treatment (acid or alkali treatments), and more sustainable alternatives such as regeneration and reuse of exhaustive materials, recycling, composting, or their treatment through bioremediation alternatives using microorganisms such as bacteria and fungi [195,289,290].

## 7. Summary

The large-scale production and use of pharmaceuticals worldwide cause the unavoidable release of different pharmaceutical pollutants into the environment, mainly in water bodies. The presence of these molecules in the environment has been recently recognized as a potential risk for ecosystems and human health. Pharmaceutical molecules in the environment are considered emerging pollutants that need regulation to reduce their environmental release and human exposure, establish their permissible concentration limits in water bodies, and avoid their ecotoxicological adverse effects on aquatic organisms, such as oxidative stress induction, genotoxicity, cytotoxicity, endocrine disruption, teratogenic effects, and reductions in growth and reproduction rates being among the most important.

Different alternatives have been proposed for eliminating pharmaceuticals from aquatic systems, including chemical precipitation, advanced oxidation processes, ionic exchange, electrochemical methods, membrane filtering, biodegradation, and biosorption through different adsorbing materials. Among these, non-conventional materials such as lignocellulosic materials generated as waste during agroindustry production processes and biochar have become relevant due to their high availability, low cost, and, most importantly, their high versatility and removal efficiency. Biochar can be derived from various agro-industrial wastes, offering a wide range of potential applications.

In the present review, 94 recent studies (2012–2024) on using residual lignocellulosic materials to remove pharmaceutical pollutants from water were analyzed. The leading pharmaceutical pollutants included in these studies were antibiotics (sulfamethoxazole and tetracycline) and NSAIDs (diclofenac, ibuprofen, and acetaminophen). However, the removal of multiple drugs such as antimicrobials, antiepileptics, and hormones from water was also evaluated.

The diversity in pollutant molecules, agro-industrial wastes, and experimental conditions used in the identified studies make it difficult to compare and identify waste materials for higher efficiency in removing pharmaceutical pollutants; the drug mass removal rate (DMRR) proposed in the present review could be a useful tool to compare different agro-industrial wastes and biosorbent materials in pharmaceutical pollutant removal from water.

The most representative materials used in these studies were biochars derived from different agro-industrial wastes. The chemical activation of these materials was a frequent method used to improve the removal of pharmaceutical drugs. According to the drug mass removal rate (DMRR) values calculated in the present review, the biosorbent material with the highest pharmaceutical removal potential was almond shell-activated biochar (1940 mg/g·h) for antibiotics, cork powder (10,420 mg/g·h) for NSAIDs, sugarcane bagasse (3184.5 mg/g·h) for chloroquine, and activated biochar from grapefruit peel (244.2 mg/g·h) used in the removal of carbamazepine. However, most of these materials could be generated and were highly available only in specific areas, thus limiting their use in the removal of pharmaceuticals on a broader scale. Due to this, agro-industrial wastes derived from crops with higher global distribution (e.g., corn, rice, soy, sugarcane, wheat) as raw materials for the removal of pharmaceutical molecules from water could be a better option if their removal efficiency is comparable to that of the above-mentioned materials.

The studies included in this review were conducted in batch systems to evaluate the removal of single pharmaceutical molecules; however, in aquatic ecosystems, mixtures of multiple molecules with different pharmacological applications as well as diverse ecotoxicological effects can be identified. Due to this, future trends in the evaluation of agro-industrial wastes for the removal of pharmaceutical pollutants from water could include removal studies no continuous systems and the treatment of mixtures of pharmaceutical pollutants.

The main findings of the present review suggest that biochar derived from agro-industrial wastes could make for excellent biosorbent materials to remove different pollutants of pharmaceutical origin. One of the most promising aspects of biochar is its cost-effective production, which could make pharmaceutical pollutant removal more affordable in the future. These alternative materials could replace other currently used pharmaceutical pollutant removal materials from water, offering a more cost-effective solution. The present review highlights the potential of biochar and the affordability it could bring to the research and development of efficient pharmaceutical contaminant removal systems based on the use of residual lignocellulosic materials.
